# Childhood Trauma and Experience in Close Relationships Are Associated with the God Image: Does Religiosity Make a Difference?

**DOI:** 10.3390/ijerph17238841

**Published:** 2020-11-28

**Authors:** Alice Kosarkova, Klara Malinakova, Jitse P. van Dijk, Peter Tavel

**Affiliations:** 1 Olomouc University Social Health Institute, Palacky University Olomouc, 771 11 Olomouc, Czech; klara.malinakova@oushi.upol.cz (K.M.); j.p.van.dijk@umcg.nl (J.P.v.D.); peter.tavel@oushi.upol.cz (P.T.); 2 Department of Community and Occupational Medicine, University Medical Center Groningen, University of Groningen, 9713 AV Groningen, The Netherlands; 3 Graduate School Kosice Institute for Society and Health, P.J. Safarik University in Kosice, 040 11 Kosice, Slovakia

**Keywords:** childhood trauma, experience in close relationships, image of God, religiosity

## Abstract

Religiosity and spirituality (R/S) and some of their specific aspects are associated with health. A negatively perceived relationship with God, which has adverse health outcomes, can be formed by human attachment both in childhood and adulthood. The aim of this study was to assess the associations of childhood trauma (CT) and experience in close relationships (ECR) with the God image in a secular environment by religiosity. A national representative sample of Czech adults (n = 1800, 51.1 ± 17.2 years; 43.5% men) participated in a survey. We measured CT (Childhood Trauma Questionnaire), ECR (Experiences in Close Relationships-Revised Questionnaire), image of God (questions from the 2005 Baylor Survey) and religiosity. Our results showed associations of CT and ECR with God images. Respondents who experienced CT were less likely to describe God as loving, always present and forgiving. Religious respondents were less likely to report positive God images with odds ratios (ORs) from 0.78 (0.66–0.94) to 0.95 (0.91–0.99), nonreligious respondents reported negative God images with ORs from 1.03 (1.00–1.06) to 1.22 (1.08–1.37). We found CT and problems in close relationships in adulthood are associated with a less positive God image, especially in nonreligious people. Understanding these associations may help prevent detrimental health outcomes.

## 1. Introduction

A growing body of research suggests that religiosity and spirituality (R/S) have positive associations with many aspects of people’s lives [1,2]. However, a small number of studies can be found that show opposite or mixed results [2]. Besides other possible explanations, e.g., measurement problems and sociocultural differences [3], it is also possible to consider different ways in which people experience their R/S, specifically their perceived quality of the relationship with God [4] and the image of God [5,6]. Recent research distinguishes between a God concept and a God image. A concept of God is formed by intellectual knowledge, religious education, culture, context and community that may be expressed in verbal descriptions of God [7,8]. An image of God is a subjective experience of what an individual or community perceives as God, based on the way in which a person unconsciously interacts with God at an emotional, nonverbal and often implicit level [9,10,11]. As such it is not only an intellectual reification within the mind [9] but involves the dynamics of aggregating memories from various sources and relationships, including the relationship to oneself [10], and associating them with God. Along these lines, the images of God are not important only for peoples’ religious and social outcomes [12,13] but also for their physical and mental health outcomes. Negative attitudes towards God, e.g., a fear of abandonment, feeling unforgiven or punished are related to a poorer well-being and worse health [13,14,15]. Similarly, impersonal or hostile God images, e.g., distant, cruel or unconcerned, are associated with difficulties in finding a meaning in life and comfort in difficult life situations [16], anxiety and depression [6,17], greater neuroticism [18,19] and faster disease progression [20]. These findings highlight the need for understanding under what conditions people tend to lean towards either a positive or a negative God image.

The processes of forming one’s implicit image of God may be influenced by childhood treatment in a positive but also in a negative way. A negative relationship exists between childhood maltreatment and R/S and many childhood abuse victims tend to view God rather negatively, such as unloving or distant [21,22]. So far, most of the research has been done on the connection between religiosity and childhood sexual abuse, where survivors have reported lower levels of spiritual well-being together with a disrupted sense of relationship with God or a higher power. They were less likely to feel loved and accepted by God [23,24]. Furthermore, the survivors described God using negative attributes (e.g., wrathful, judgmental, uncaring) and reported negative feelings and difficulty in accepting God’s love and kindness [25]. However, a few studies can be found that examined other forms of childhood maltreatment [22,26,27]. These are proposing that emotional, physical and verbal mistreatment also have a negative impact on religiosity and the image of God. 

Moreover, a childhood experience of mistreatment may affect later attachment relationships [28,29]. Therefore, it is possible that this experience involves also the attachment to God; that has been shown as corresponding to a human attachment [30,31] and can be described similarly [32]. Results from several studies have shown that a secure adult attachment was associated with a more positive image of God and a feeling towards God [26,33]. In contrast, avoidant attachment was related to the lack of a secure, positive relationship to God, a desire to keep God at a distance [34] and to the image of God as controlling or unavailable [33]. Similarly, anxious attachment was associated with an experience of abandonment or punishment by God as a projection of a personal attachment style [34] and with anxious attachment to God [30]. Thus, adult attachment may be likely relevant in forming one’s God image. 

Thus far, most studies on the images of God and their relation to health issues or their possible roots have been conducted outside of Europe [35,36,37,38] and only a very few within Europe [16,39,40,41]. The Czech Republic is according to some sources one of the most secular countries in the world with the highest percentage (76.4%) of religiously unaffiliated people [42], and it is characterized by a high degree of secularization, as most people do not report any religious affiliation or regular church attendance [43]. This represents a unique setting to assess the images of God and the inclination to draw on them. 

Therefore, the aim of this study was to assess the associations of childhood trauma and anxiety and avoidance in adult attachment with God images among Czech adults in a secular environment taking into account one’s self-reported religiosity.

## 2. Materials and Methods 

### 2.1. Participants and Procedure 

We obtained a national sample of the Czech population of fifteen years old and over, which was acquired by using a two-step procedure. Having piloted the questionnaire and all further procedures on 206 participants, the final version of the survey was developed. In the second step, another 2184 random participants were chosen with the help of quota sampling and were asked to participate in a study on the problems of health, life experiences, attitudes and lifestyle. Quota sampling is a technique often used in research to imitate the known characteristics of the population in the sample, allowing relationships between subgroups to be observed. In this case the criteria that allowed the construction of a representative sample corresponding to the adult Czech population were used. Of these respondents, 384 (17.6%) refused to participate in the survey. The participants reported a lack of time (39.2%), a lack of interest in or distrust in research in general (24.0%), the personal nature of the questions (17.2%) and the length and difficulty of the questionnaire (11.2%) among the main reasons for refusal. The final sample consisted of 1800 respondents. 

The data was collected by professionally trained administrators in September and October 2016 during a structured interview with the respondents. The participants received written information on the aim of the study and the anonymized handling of the data and were made familiar with the system. Participation in the survey was fully voluntary; respondents did not receive compensation for their participation in the survey, so they could stop responding to the survey at any time before or during the interview. Therefore, starting the survey was seen as providing informed consent. The study design was approved by the Ethics Committee of the Olomouc University Social Health Institute, Palacky University in Olomouc (No. 2016/3).

### 2.2. Measures 

All instruments were available in the Czech language. 

Image of God was assessed using 18 adjectives describing God preceded by the question “How well do you feel that each of the following words describe God?”. Of these adjectives (e.g., critical, distant, loving, just), 15 were taken from the 2005 Baylor Religion Survey (Baylor University, Waco, TX, USA, 2005). Respondents chose from the possible answers “very well” (1); “somewhat well” (2); “not very well” (3); “not at all (4)”. The respondents who identified themselves as believers and were further considered as religious described how well in their opinion the adjectives describe God. The respondents who did not identify themselves as believers were considered nonreligious and as such they were asked how well, according to them, these adjectives describe the opinion of religious respondents. This approach was chosen because nonreligious respondents could not be asked directly about God’s characteristics. However, their responses can still offer a certain image of God, who they do not believe in [44]. For the purpose of statistical analysis, each item was dichotomized following the approach of Malinakova et al. [3]. Therefore, only the respondents from both the religious and nonreligious groups who declared a full agreement/disagreement with a specific adjective were considered as seeing God in this way. This means that for the positive adjectives, only the response option (1) “very well” was coded as 1, while for the negative adjectives these were all the options with the exception of (4) “not at all”. There were 11 positive and 7 negative adjectives altogether. 

To assess the experience in close relationships, a shortened version of the Experiences in Close Relationships-Revised (ECR-R-16) questionnaire was used [45]. It is composed of 16 items that measure two dimensions of an attachment-related experience. It was validated for the Czech environment [46,47]. The questionnaire is split into two subscales, with each subscale consisting of 8 items. The respondents could choose from possible answers ranging from “totally disagree” (1) to “totally agree” (7), where (4) allows one to choose a neutral response, resulting in scores from 8 to 56 for each subscale. The Anxiety subscale measures the extent to which people are insecure about the availability and responsiveness of a romantic partner. The Avoidance subscale measures the extent to which people feel uncomfortable being close to others. In the main analyses, both subscales were assessed as a binary variable created by dichotomizing the score with the subscale’s upper quartile as the cut-off point, as used, e.g., in [3]. Cronbach’s alpha in our sample was 0.85. 

Childhood trauma was assessed using the Childhood Trauma Questionnaire (CTQ) [48]; validated for Czech conditions [49]. The CTQ is a standardized 28 item inventory, which was developed to assess the importance of five types of abuse and maltreatment experienced in childhood or adolescence. The CTQ consists of five subscales: Emotional Abuse, Physical Abuse, Sexual Abuse, Emotional Neglect and Physical Neglect. Each of the subscales consists of five items rated on a 5-point Likert-type scale. Thus, our respondents could choose from answers ranging from “never” (1) to “very often” (5), resulting in scores from 5 to 25 for each subscale. Cronbach’s alpha for the CTQ subscales in our sample ranged from 0.62 to 0.89. 

Religiosity was measured with the question: “At present, would you call yourself a believer?” with possible answers: “yes, I am a member of a church or religious society”; “yes, but I am not a member of a church or religious society”; “no”; “no, I am a convinced atheist”. For the purpose of further analysis, participants who reported “yes” were dichotomized as religious.

Gender, age, education, marital status, living arrangements and economic activity were obtained through the questionnaire.

### 2.3. Statistical Analyses 

First, we described the background characteristic of the sample. Because of the non-normal distribution of the data, nonparametric methods were used to compare different sociodemographic groups as well as for the main analyses. In the next step, the associations of childhood trauma subscales (standardized to z-scores) with different adjectives describing a God image were assessed using a binary logistic regression model adjusted for gender, age and education. The respondents were divided into groups according to their religiosity. Further, the same process was repeated for the associations of anxiety and avoidance in a close relationship. Each independent variable was tested in a separate model. All analyses were performed using the statistical software package IBM SPSS version 25 (IBM Corp., Armonk, NY, USA). 

## 3. Results

### 3.1. Description of the Population 

The background characteristics of the sample (mean age 46.4, SD = 17.4; 95%CI = 45.60–47.21; 48.7% men) are presented in Table 1. The sample is a sample of the Czech population over 15 years of age. Of the whole sample, 29.5% of the respondents labelled themselves as religious; 5.8% of the whole sample regularly attended religious services. 

### 3.2. Specific Images of God with Childhood Trauma and Experience in a Close Relationship

Table 2 shows the associations of the specific images of God with CT and ECR. Some God images showed no significant associations, like demanding, kingly or punishing. In associations with CT, the nonreligious respondents were less likely to describe their God image (i.e., the way in which in their opinion religious respondents see God) as absolute or fatherly and they were more likely to see him more critical than it was expressed by religious respondents. Similarly, only the nonreligious with a higher attachment avoidance described their image of God as more critical, serious and angry. Nevertheless, attachment avoidance was negatively associated with a positive God image in both studied groups. We found no significant associations of both CT and ECR with a distant, kingly, punishing, unpredictable or demanding God image. The strongest associations were found for an absolute God image and physical neglect, odds ratio (OR) 0.75 (95% confidence interval, CI, 0.65–0.86) and for an always present image of God with physical neglect, OR 0.72 (95% CI 0.63–0.82), both within the group of nonreligious respondents. Furthermore, the most frequent associations were found for the loving image of God, where a one SD increase in emotional neglect was associated with a 5% decrease in the odds of seeing God this way and one SD increase in physical neglect in nonreligious respondents with even an 11% decrease in the same odds. In the religious group we found negative associations between the positive adjectives such as forgiving, loving or always present and CT and ECR.

## 4. Discussion

The aim of this study was to assess the associations of childhood trauma and adult attachment with God images in a highly secular environment taking into account one’s self-reported religiosity. We found that both the religious and nonreligious respondents who experienced any kind of childhood trauma were less likely to describe God as loving, always present and forgiving. Similarly, those who reported anxiety or avoidance in a close relationship were less likely to describe God as forgiving or just. Furthermore, the nonreligious respondents who experienced a childhood trauma were less likely to report God as absolute or fatherly and more likely to describe God as critical.

We found that the participants who reported some kind of childhood trauma were less likely to report positive images of God. They hesitated to describe God as loving, always present, forgiving, fatherly or just and rather used terms such as critical or angry. In line with the findings of other authors [27,50,51], it may be assumed that survivors of a childhood trauma experience a negative self-perception, feelings of shame and being unworthy and that they transmit their negative feelings to a spiritual dimension [52]. The victims’ sense of being loved and accepted by God can be disrupted [53], and they can have difficulty in believing in God’s love [54,55]. Furthermore, they may question God’s power and justice [56,57,58] and underreport God as absolute or just.

However, we did not find significant associations between childhood trauma and a distant and punishing God image. Thus, our findings are in contrast to those of other authors, who associated a distant and controlling image of God with sexual abuse [22] and with other forms of maltreatment [26,27]. It could be argued that in some cases an experienced trauma might have led to increased spirituality as some studies suggest on post-traumatic spiritual growth [59] and acquiring a positive God image helps survivors during their process of recovery and their ability to cope with the history of the trauma [23,26]. Moreover, the positive image of God may operate in a compensatory manner and fulfil the victims’ search for security and a safe haven [7,31,60,61]

Furthermore, our results showed significant associations between interpersonal avoidance and less loving, fatherly, forgiving and always present God images. These findings are again in contrast with other research results [32,33] in which the authors suggested that highly avoidant people can regulate their distress from human relationship difficulties by turning to a relationship with God, who could fulfil their desires and forgive trespasses. However, our results are in line with other studies that showed that an insecure human relationship strengthens negative perceptions of God [33] and found negative correlations between a loving God image and avoidance and a positive association with a controlling image [30]. Moreover, as God can be seen as an attachment figure [62], we may argue that an insecure adult attachment corresponds with an insecure attachment to God [60,63]. Avoidant respondents may mirror their interpersonal relationship experience in their relation to God [7] and thus in describing God’s image they associate their fear of being dependent on a partner with adjectives that express their insecure attitudes.

We further found that participants who described God as critical, serious or angry were more likely to experience anxiety in close relationships. These results further support the correspondence of anxiety in an interpersonal relationship to anxiety in relation to God [33,60]. It could be supposed that a person with relationship anxiety feels unworthy and in need of self-approval from their partner. Thus, they can transmit these feelings towards God [34] and experiencing insufficiency and uncertainty can lead to viewing God rather negatively.

We observed different patterns in the associations between the groups of religious and nonreligious respondents. In general, the nonreligious respondents expected among the religious a more negative image of God than the religious respondents reported. The images of God as less absolute, kind, generous and less motherly but more critical and serious were referred to in nonreligious respondents but not so in the religious. Moreover, the religious respondents did not report less positive and more negative images of God as much as the nonreligious did. These findings are in line with the studies which describe that though some religious respondents can see God as distant [64] and cruel [65], they do not report these feelings so strongly as they report a loving God image [38,44]. This opens the possibility that religious respondents may have been reluctant to report negative attitudes towards God using the negative adjectives; they might fear having doubts about God or expressing negative attitudes could bring punishment and be morally unacceptable [66,67]. Instead, they may rather report positive images to somehow protect their God image in a nonreligious environment [44].

### 4.1. Strengths and Limitations

This study has some important strengths. The main strength is that it is based on a representative sample with a high response rate. Furthermore, the completed questionnaires had no missing values. It is also one of the few studies that assess the associations of the images of God with an adult attachment and a childhood trauma experience in a highly secular environment. However, though the study contributed to the deeper understanding of God images, it also has several limitations. The first is the cross-sectional design of the study, which does not allow us to make causal inferences. Additionally, since our data were self-reported, religiously affiliated respondents might have responded according to their religious education and thus provided socially desirable responses. Moreover, as religiosity and spirituality can be seen as different concepts, we consider the fact we did not assess them separately, as a limitation and further studies should focus on this. Another limitation is that we did not consider all genders but only men and women. However, gender differences were not the main focus of this study. Thus, we assume that this did not influence the validity of the study. Furthermore, as the other sociodemographic variables were used as covariates only, the limited attention to these contextual elements can be considered another limitation of the present study. Last but not least, it must also be mentioned as a limitation the way the term “image of God” is used since different cultures, religions and contexts may use different concepts followed by quite different expressions. These limitations should be included in follow-up studies in order to achieve a more precise understanding of associations between insecure attachment and the images of God.

### 4.2. Implications

Our findings suggest that attachment avoidance and anxiety as well as a childhood trauma experience may negatively affect an adult’s image of God. Understanding these associations might therefore be important for professional counselling interventions in the area of spirituality or care. Furthermore, the results contribute to widening the range of factors that help those experiencing and dealing with trauma. At the same time, our results also show that using a negative and/or lower usage of positive God’s images can serve as a sign of attachment insecurity and distress and, therefore, may be informative for professionals in other areas, such as psychotherapy, psychosomatic medicine or social work, where internalization of spiritual values can help the effectiveness of the interventions.

Further research is needed to explore the influence of both the partner’s and parents’ religiosity on the development of one’s image of God. In addition, the role of a perpetrator of violence should be further considered. Moreover, further research should focus on the representations of God in different religions and distinguish between the person-like terms of the Christian tradition, the more philosophical Jewish terms of an unimaginable God [68] and the Muslim ban on anthropomorphizing God. Thus, future research on this topic and on the causal pathway is recommended. Furthermore, since this study has a cross-sectional design, further studies should focus on the causal effects of the image of God developed as a consequence of the childhood traumatic experience and on the mutual interaction between images of God and a life of a secular society.

## 5. Conclusions

Our findings suggest that childhood trauma and adult attachment are associated with a less positive God image. Individuals with an experience of a childhood trauma tend to view God in more negative terms and hesitate to use positive terms. The same applies to the respondents with an experience of relationship anxiety or avoidance. Furthermore, different patterns were found between religious and nonreligious respondents. The religious respondents reported less negative and more positive images of God than the nonreligious did. Moreover, the nonreligious respondents expected among the religious more negative images and referred to the images of God as less absolute, kind and generous but more critical and serious. Thus, this study offers a deeper understanding of the factors, which may contribute to the forming of one’s God image and which may further lead to the use of maladaptive religious coping strategies, inviting further research to clarify these associations.

## Figures and Tables

**Table 1:** Description of the study population, total and by religiosity.

Characteristics	Total		Nonreligious		Religious	
	N	%	N	%	N	%
**Gender**						
Male	877	48.7	646	50.9	231	43.5
Female	923	51.3	623	49.1	300	56.5
**Age**						
15–29 years old	410	22.8	324	25.5	86	16.2
30–49 years old	619	34.4	465	36.6	154	29.0
50–69 years old	588	32.7	379	29.9	209	39.4
70–90 years old	183	10.2	101	8.0	82	15.4
**Living arrangement**						
With husband/wife	921	51.2	616	48.5	305	57.4
With unmarried mate	351	19.5	284	22.4	67	12.6
Alone	353	19.6	237	18.7	116	21.8
With parents/siblings	175	9.7	132	10.4	43	8.1
**Marital status**						
Single/ Divorced/ Widow-widower	730	40.6	531	41.8	199	37.5
Married/ Partner relationship	1070	59.4	738	58.2	332	62.5
**Highest education achieved**						
Elementary school	141	7.8	104	8.2	37	7.0
Secondary vocational school	442	24.6	292	23.0	150	28.2
Secondary school with graduation	854	47.4	620	48.9	234	44.1
College	363	20.2	253	19.9	110	20.7
**Economic activity**						
Employee	939	52.2	698	55.0	241	45.4
Self-employed	170	9.4	125	9.9	45	8.5
In the household ^a^/Unemployed	83	4.6	56	4.4	27	5.1
Student	178	9.9	139	11.0	39	7.3
Disabled/Old-age pensioner	430	23.9	251	19.8	179	33.7
**Total**	1800	100	1269	70.5	531	29.5

Note: ^a^ including maternity leave.

**Table 2:** Associations of different childhood trauma experiences and the experiences in close relationships (both standardized to z-scores) with different images of God: results of binary logistic regression adjusted for age, gender and education level leading to odds ratios (OR) with 95% confidence intervals (95% CI).

Variables	Absolute	Critical	Distant		Always Present	Fatherly	Forgiving
**Childhood trauma experience**							
Emotional abuse	NR ^1^	0.94 (0.82–1.07)	1.20 (1.05–1.36) **	1.02 (0.90–1.15)	0.89 (0.78–1.00)	0.93 (0.82–1.05)	0.92 (0.81–1.04)
	R ^2^	1.10 (0.92–1.31)	1.01 (0.83–1.22)	1.02 (0.84–1.23)	0.94 (0.79–1.12)	0.98 (0.82–1.16)	0.93 (0.78–1.11)
Physical abuse	NR	0.90 (0.79–1.04)	1.19 (1.04–1.37) *	1.03 (0.90–1.16)	0.90 (0.80–1.03)	0.93 (0.81–1.05)	0.92 (0.81–1.04)
	R	1.11 (0.93–1.32)	1.06 (0.87–1.30)	1.01 (0.83–1.22)	0.93 (0.78–1.10)	0.98 (0.83–1.17)	0.92 (0.77–1.09)
Sexual abuse	NR	0.79 (0.66–0.94) **	1.14 (1.00–1.30)	1.00 (0.88–1.13)	0.78 (0.66–0.91) **	0.84 (0.72–0.98) *	0.75 (0.64–0.89) **
	R	0.90 (0.74–1.10)	1.08 (0.88–1.33)	1.20 (0.93–1.54)	0.83 (0.69–0.99) *	0.92 (0.77–1.09)	0.82 (0.68–0.99) *
Emotional neglect	NR	0.86 (0.75–0.98) *	1.22 (1.08–1.37) **	1.08 (0.95–1.23)	0.84 (0.74–0.94) **	0.85 (0.75–0.96) *	0.90 (0.80–1.02)
	R	0.96 (0.80–1.15)	0.93 (0.77–1.12)	1.12 (0.92–1.36)	0.89 (0.75–1.07)	0.88 (0.74–1.04)	0.81 (0.68–0.97) *
Physical neglect	NR	0.75 (0.65–0.86) ***	1.18 (1.05–1.34) **	1.13 (0.99–1.29)	0.72 (0.63–0.82) ***	0.79 (0.69–0.90) **	0.86 (0.76–0.97) *
	R	1.01 (0.87–1.25)	1.16 (0.94–1.42)	1.22 (0.99–1.49)	0.85 (0.71–1.02)	0.94 (0.79–1.13)	0.92 (0.87–0.98) *
**Experience in a close relationship**							
Anxiety	NR	0.92 (0.81–1.04)	1.16 (1.03–1.31) *	1.12 (0.99–1.28)	0.93 (0.83–1.05)	0.90 (0.79–1.02)	0.94 (0.83–1.06)
	R	0.80 (0.67–0.97) *	1.18 (0.96–1.43)	1.21 (0.99–1.48)	0.97 (0.81–1.15)	0.97 (0.81–1.15)	0.80 (0.67–0.96) *
Avoidance	NR	0.91 (0.80–1.03)	1.00 (0.89–1.13)	1.10 (0.97–1.25)	0.86 (0.76–0.97) *	0.80 (0.71–0.91) **	0.83 (0.74–0.94) **
	R	0.88 (0.73–1.05)	1.01 (0.84–1.23)	1.22 (1.00–1.48)	0.79 (0.66–0.94) **	0.82 (0.69–0.98) *	0.75 (0.63–0.90) **
		**Friendly**	**Just**	**Kind**	**Kingly**	**Loving**	**Motherly**
**Childhood trauma experience**							
Emotional abuse	NR	0.95 (0.91–0.99) *	0.95 (0.91–0.99)	0.97 (0.92–1.02)	1.02 (0.98–1.07)	0.95 (0.91–1.00) *	0.99 (0.94–1.03)
	R	0.96 (0.90–1.02)	0.99 (0.93–1.05)	0.97 (0.90–1.04)	0.99 (0.93–1.06)	0.95 (0.89–1.01)	0.97 (0.90–1.03)
Physical abuse	NR	0.98 (0.92–1.04)	0.97 (0.92–1.03)	0.94 (0.87–1.01)	1.01 (0.94–1.06)	0.95 (0.90–1.01)	0.95 0.89–1.02)
	R	0.99 (0.90–1.08)	0.98 (0.90–1.07)	1.04 (0.95–1.14)	0.96 (0.88–1.06)	0.95 (0.87–1.03)	1.02 (0.93–1.11)
Sexual abuse	NR	0.89 (0.82–0.97) **	0.96 (0.90–1.1.03)	0.98 (0.91–1.06)	0.99 (0.93–1.06)	0.92 (0.8–0.98) *	0.87 (0.87–1.01)
	R	0.86 (0.75–0.97) *	0.88 (0.78–0.99) *	0.96 (0.85–1.08)	0.89 (0.77–1.02)	0.86 (0.77–0.97) *	0.95 (0.85–1.07)
Emotional neglect	NR	0.98 (0.96–1.01)	0.99 (0.96–1.01)	0.97 (0.94–1.00) *	0.99 (0.96–1.02)	0.95 (0.93–0.98) ***	0.97 (0.94–0.99) *
	R	0.97 (0.93–1.01)	0.95 (0.91–0.99) *	0.98 (0.93–1.02)	0.98 (0.94–1.02)	0.95 (0.91–0.99) *	0.97 (0.93–1.01)
Physical neglect	NR	0.96 (0.92–1.01)	0.94 (0.90–0.98) **	0.94 (0.89–1.00) *	0.98 (0.93–1.04)	0.89 (0.85–0.94) ***	0.92 (0.87–0.97) **
	R	0.97 (0.91–1.04)	0.96 (0.90–1.02)	1.00 (0.94–1.07)	0.98 (0.92–1.05)	0.94 (0.88–1.00) *	0.99 (0.93–1.05)
**Experience in a close relationship**							
Anxiety	NR	0.96 (0.85–1.09)	0.88 (0.78–0.99) *	0.93 (0.80–1.07)	0.98 (0.85–1.12)	0.90 (0.80–1.01)	0.88 (0.77–1.00)
	R	0.86 (0.72–1.02)	0.82 (0.69–0.97) *	0.84 (0.69–1.01)	0.91 (0.76–1.09)	0.90 (0.75–1.07)	0.88 (0.73–1.05)
Avoidance	NR	0.94 (0.83–1.06)	0.92 (0.82–1.04)	1.08 (0.94–1.24)	0.95 (0.83–1.09)	0.83 (0.74–0.94) **	0.89 (0.78–1.02)
	R	0.78 (0.66–0.94) **	0.82 ((0.68–0.97) *	1.03 (0.86–1.24)	0.93 (0.78–1.11)	0.78 (0.65–0.93) **	0.90 (0.76–1.08)
		**Punishing**	**Serious**	**Angry**	**Generous**	**Unpredictable**	**Demanding**
**Childhood trauma experience**							
Emotional abuse	NR	1.04 (0.99–1.09)	1.02 (0.98–1.07)	1.02 (0.98–1.07)	0.99 (0.94–1.03)	1.04 (1.00–1.09)	1.05 (1.01–1.10) *
	R	1.03 (0.95–1.13)	1.03 (0.94–1.13)	1.07 (0.99–1.15)	1.01 (0.95–1.08)	1.00 (0.94–1.08)	1.02 (0.95–1.09)
Physical abuse	NR	1.02 (0.96–1.08)	1.00 (0.95–1.06)	1.01 (0.96–1.07)	0.98 (0.93–1.04)	1.01 (0.96–1.06)	1.07 (1.00–1.13)
	R	1.07 (0.94–1.22)	1.06 (0.93–1.21)	1.07 (0.97–1.19)	1.04 (0.95–1.14)	1.04 (0.94–1.14)	0.98 (0.89–1.08)
Sexual abuse	NR	0.99 (0.93–1.05)	0.96 (0.91–1.02)	1.01 (0.95–1.07)	0.98 (0.92–1.04)	1.03 (0.97–1.09)	0.99 (0.94–1.05)
	R	1.17 0.94–1.47)	1.09 (0.89–1.33)	1.09 (0.95–1.25)	0.97 (0.86–1.08)	1.04 (0.91–1.18)	0.96 (0–86-1.07)
Emotional neglect	NR	1.02 (0.99–1.04)	1.02 (1.00–1.05)	1.03 (1.00–1.06) *	0.98 (0.95–1.01)	1.02 (1.00–1.05)	1.02 (0.96–1.05)
	R	1.01 (0.96–1.07)	1.04 (0.98–1.10)	1.08 (1.03–1.13) **	0.96 (0.92–1.00)	1.01 (0.97–1.06)	1.02 (0.97–1.07)
Physical neglect	NR	0.99 (0.94–1.04)	0.99 (0.94–1.04)	1.02 (0.97–1.06)	0.93 (0.88–0.98) **	1.04 (0.99–1.09)	1.02 (0.97–1.07)
	R	1.07 (0.98–1.16)	1.04 (0.95–1.14)	1.14 (1.06–1.23) **	0.94 (0.89–1.01)	1.04 (0.97–1.11)	1.02 (0.95–1.09)
**Experience in a close relationship**							
Anxiety	NR	1.11 (0.97–1.26)	1.19 (1.04–1.36) *	1.18 (1.04–1.34) **	0.95 (0.84–1.08)	1.10 (0.98–1.24)	1.11 (0.98–1.26)
	R	1.23 (0.97–1.56)	1.23 (0.96–1.59)	1.19 (0.99–1.44)	0.82 (0.69–0.98) *	1.12 (0.93–1.36)	1.09 (0.90–1.32)
Avoidance	NR	0.92 (0.81–1.04)	0.93 (0.82–1.05)	1.04 (0.92–1.18)	0.90 (0.79–1.03)	1.07 (0.95–1.20)	1.01 (0.90–1.14)
	R	1.14 (0.91–1.43)	1.00 (0.79–1.26)	1.15 (0.95–1.38)	0.86 (0.72–1.03)	1.04 (0.86–1.25)	1.07 (0.89–1.30)

Notes: ^1^ nonreligious, ^2^ religious; * *p* < 0.05, ** *p* < 0.01, *** *p* < 0.001; SD—standard deviation.
